# Arterial pressure changes monitoring with a new precordial noninvasive sensor

**DOI:** 10.1186/1476-7120-6-41

**Published:** 2008-08-21

**Authors:** Tonino Bombardini, Vincenzo Gemignani, Elisabetta Bianchini, Lucia Venneri, Christina Petersen, Emilio Pasanisi, Lorenza Pratali, Mascia Pianelli, Francesco Faita, Massimo Giannoni, Giorgio Arpesella, Eugenio Picano

**Affiliations:** 1 Department of Echocardiography Lab, Fondazione Gabriele Monasterio, Italian National Research Council, Pisa, Italy; 2 Digital Signal Processing Lab (DSPLAB), Fondazione Gabriele Monasterio, Italian National Research Council, Pisa, Italy; 3 Department of Surgery and Transplants, University of Bologna, Italy

## Abstract

**Background:**

Recently, a cutaneous force-frequency relation recording system based on first heart sound amplitude vibrations has been validated. A further application is the assessment of Second Heart Sound (S2) amplitude variations at increasing heart rates. The aim of this study was to assess the relationship between second heart sound amplitude variations at increasing heart rates and hemodynamic changes.

**Methods:**

The transcutaneous force sensor was positioned in the precordial region in 146 consecutive patients referred for exercise (n = 99), dipyridamole (n = 41), or pacing stress (n = 6). The curve of S2 peak amplitude variation as a function of heart rate was computed as the increment with respect to the resting value.

**Results:**

A consistent S2 signal was obtained in all patients. Baseline S2 was 7.2 ± 3.3 m*g*, increasing to 12.7 ± 7.7 m*g *at peak stress. S2 percentage increase was + 133 ± 104% in the 99 exercise, + 2 ± 22% in the 41 dipyridamole, and + 31 ± 27% in the 6 pacing patients (p < 0.05). Significant determinants of S2 amplitude were blood pressure, heart rate, and cardiac index with best correlation (R = .57) for mean pressure.

**Conclusion:**

S2 recording quantitatively documents systemic pressure changes.

## Introduction

Recently, a cutaneous operator independent force-frequency relation recording system as been validated in the stress echo lab, based on first heart sound amplitude variations at increasing heart rates [1,2]. Contractility quantification and systolic/diastolic time measurement through the system has been previously demonstrated [1,3]. A further application could be the assessment of Second Heart Sound (S2) amplitude variations at increasing heart rates. In fact, the maximum amplitude of vibrations measured by the sensor following the ECG T wave originates from the physical phenomenon of the abrupt deceleration of the moving aortic blood mass. The audible components of this deceleration give rise to the Second Heart Sound (S2) [4-6]. The aim of this study was to assess the relationship between second heart sound amplitude variations at increasing heart rates and hemodynamic changes.

## Methods

### Patient selection

We enrolled 146 consecutive patients (99 males, 60 ± 14 years) referred for stress echocardiography. Patients' characteristics are summarized in Table 1. The type of stressor was chosen by the attending cardiologist/echocardiographist at time of testing in relation to relative contraindications of one stressor over the other [7,8]. Ninety-nine subjects underwent exercise stress (13 non competitive athletes were the controls). Twenty-four patients unable to exercise and 17 patients scheduled for coronary flow reserve evaluation underwent dipyridamole stress echo. Six patients with permanent pace maker (DDD in 5, BIV in 1) underwent pacing stress. Coronary artery disease was defined by the presence of angiographically assessed coronary stenosis (with quantitatively assessed diameter reduction in major coronary vessels) or previous myocardial infarction. The local Ethical Committee approved the study protocol. All patients gave their written informed consent before entering the study. All patients met the following inclusion criteria: 1) referred to stress echo for clinically-driven testing. 2) acoustic window of acceptable quality 3) willingness to enter the study. From the initially considered population of 152 patients, 4 were excluded for poor acoustic window (n = 4), or refusal to give written informed consent (n = 2).

**Table 1:** Characteristics of the study patients

	EXERCISE	DIP	PACING
Pt n°	99	41	6
Age (years)	56 ± 14	68 ± 11	68 ± 10
Males	68	27	4
Controls	13	-	-
CAD	36	29	3
Previous PTCA/By pass	27	19	1
Previous myocardial infarction	25	13	2
Arterial hypertension	18	5	-
Valvular disease	19	2	1
Atipical chest pain	12	3	1
DCM	1	2	1

### Semi-supine bicycle exercise

Graded bicycle semi-supine exercise echo was performed starting at an initial workload of 25 watts lasting for 2 minutes; thereafter the workload was increased stepwise by 25 watts at 2 minutes interval. A 12-lead electrocardiogram and blood pressure determination were performed at baseline and every minute thereafter [7]. Two-dimensional echocardiographic monitoring was performed throughout and up to 5 min after the end of peak stress. Two-dimensional images were recorded at baseline and at the end of each step.

### Dipyridamole stress echo

Two-dimensional echocardiography and 12-lead electrocardiographic (ECG) monitoring were performed in combination with high dose dipyridamole (up to 0.84 mg over 6 min) in accordance to well established protocols [7,8]. Contraindications to using dipyridamole were asthma, hypotension, and bradyarrhythmias.

### Pacing stress echo

The pacing protocol was accelerated (with a 10-beat increment every 60 s) until one of the following criteria was reached: 1 – 85% of maximal heart rate (age-corrected: 220 – age for men, 200 – age for women); or 2 – PM maximal programmable heart rate (which varied widely, according to the model of PM, up to 170 bpm during stress). Stimulation was performed, wherever possible, in atrial stimulation mode, or dual-chamber (DDD) pacing to have normal contraction sequence [9].

### Regional wall motion analysis

Regional wall motion analysis was evaluated at baseline and at peak stress with a semiquantitative assessment of a wall motion score index (WMSI), with the 17 segment model of the left ventricle, each segment ranging from 1 = normal/hyperkinetic to 4 = dyskinetic, according to the recommendations of the American Heart Association and American Society of Echocardiography. WMSI was derived by dividing the sum of individual segment scores by the number of interpretable segments [8,10]. Test positivity was defined as the occurrence of at least one of the following conditions: 1) new dyssynergy in a region with normal rest function (i.e., normokinesia becoming hypokinesia, akinesia or dyskinesia) in at least two adjacent segments.

### Diagnostic end points and interruption criteria

The diagnostic end-points for all types of stress were: the development of obvious echocardiography positivity. Non-echocardiographic test end-points were the following: peak dipyridamole dose; 85% of target heart rate; achievement of conventional end-points (such as severe chest pain and/or diagnostic ST segment changes). The test was also stopped, in the absence of diagnostic endpoints, for one of the following reasons of constituting a submaximal, non-diagnostic test: intolerable symptoms; limiting asymptomatic side effects, consisting of: a) hypertension (systolic blood pressure >220 mmHg; diastolic blood pressure >120 mmHg); b) hypotension (relative or absolute): >30 mmHg fall of blood pressure; c) supraventricular arrhythmias: supraventricular tachycardia or atrial fibrillation; d) ventricular arrhythmias: ventricular tachycardia; frequent, polymorphous premature ventricular beats [8].

### Blood pressure analysis

One nurse recorded blood pressures at rest and during each individual study. The blood pressure recording was made using a sphygmomanometer and the diaphragm of a standard stethoscope. Systolic and diastolic blood pressure was obtained in the right arm. During exercise test, blood pressure recording was obtained with patient lying in a left rotated semi supine position and instructed to hand grip to the left support with their left hand. Patients have been told to let their right hand go limp when blood pressure was measured.

By selection, 75 out of the 99 patients of the exercise group had simultaneous S2 amplitude and systemic blood pressure measurement at the first, third and fifth post exercise minute time.

### Volume analysis

All patients underwent transthoracic echocardiography at baseline and during stress. Left ventricular end-diastolic and end-systolic volumes were measured from apical four- and two-chamber view, by an experienced observer using the biplane Simpson-method. Only representative cycles with optimal endocardial visualization were measured and the average of three measurements was taken. The endocardial border was traced, excluding the papillary muscles. The frame captured at the R wave of the ECG was considered to be the end-diastolic frame, and the frame with the smallest left ventricular cavity the end systolic frame. Images were acquired at baseline and at each increase in heart rate of 10 beats during stress.

### Systemic Vascular Resistance (SVR)

SVR were calculated according to the traditional formula:

SVR = 80 * (MAP-5)/CO,

where 5 is an approximation of the right atrial pressure and MAP is mean arterial pressure.

### Systemic arterial compliance

Systemic arterial compliance (C) was calculated as stroke volume index/systemic arterial pulse pressure; were pulse pressure = systolic blood pressure – diastolic blood pressure [11].

### Arterial elastance and ventricular-arterial coupling

In all, ventricular arterial coupling was indexed by the ratio of left ventricular systolic elastance index (systolic pressure/end-systolic volume index) to arterial elastance (Ea, ratio of end-systolic pressure by stroke volume). Echocardiography (for ESV and stroke volume) and cuff sphygmomanometer (systolic pressure, multiplied × 0.90 to obtain end-systolic pressure) provided the raw measurements.

Because stroke volume (and input impedance) varies directly with body size, arterial elastance was adjusted for body surface area (EaI) to better reflect differences in arterial properties with age and between the genders adjusted for differences in body size [12]. Of note ventricular-arterial coupling is ventricular elastance/arterial elastance, which can further be described as: end-systolic pressure/end-systolic LV volume divided by end-systolic pressure/stroke volume: the pressure terms in the numerator and the denominator cancel out, and ventricular-arterial coupling equals to stroke volume/end-systolic volume.

### Operator-independent second heart sound quantification

**Figure 1:**
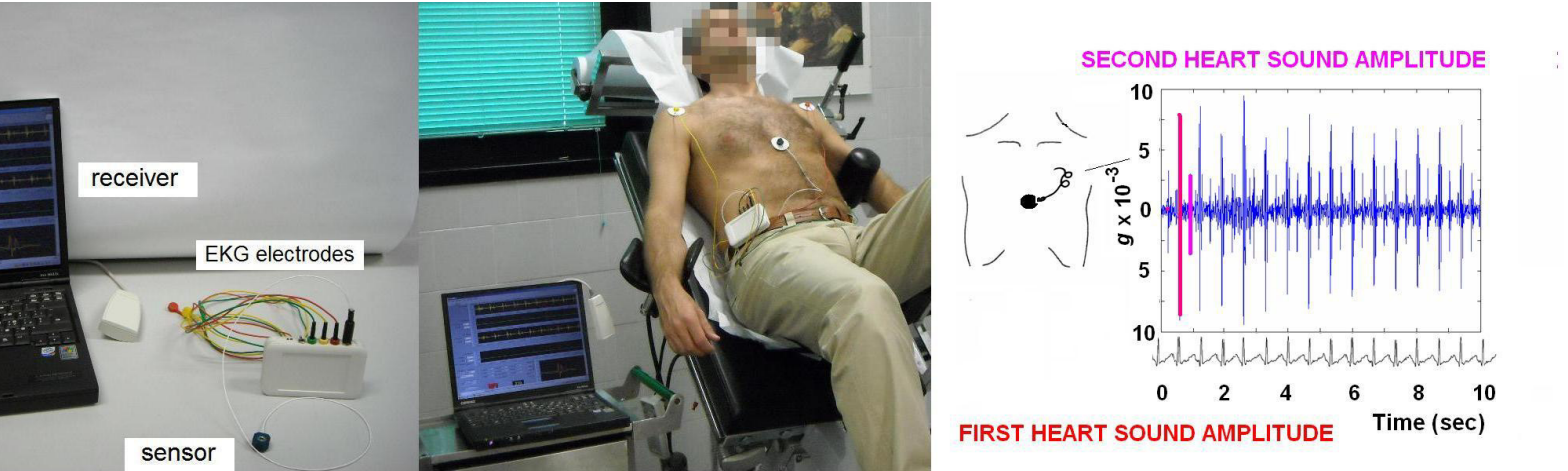
**Isovolumic contraction force and second heart sound (S2) amplitude**. A Micro-Electro-Mechanical Systems (MEMS) accelerometer is temporarily positioned in the mid-sternal precordial region before starting the scheduled stress test in all patients. A peak detection algorithm, synchronized with the ECG, scans the first 150 ms following the R wave to record the isovolumic contraction force vibration and then the interval before the following R wave to record the second heart sound amplitude (S2, pink symbol). All the parameters are acquired as instantaneous values at baseline and during stress. The data can be also read remotely by a wireless bluetooth sensor network, with reliable continuous remote monitoring 'See additional file 1: Appendix'.

The transcutaneous force sensor is based on a linear accelerometer from STMicroelectronics (LIS3). The device includes in one single package a MEMS sensor that measures a capacitance variation in response to movement or inclination and a factory trimmed interface chip that converts the capacitance variations into analog signal proportional to the motion. The device has a full scale of ± 2·*g *(*g *= 9.8 m/s^2^) with a resolution of 0.0005·*g*. We housed the device in a small case (Figure 1) which was positioned in the mid-sternal precordial region and was fastened by a solid gel ECG electrode. The acceleration signal was converted to digital and recorded by a laptop PC, together with an ECG signal. The system is also provided with a user interface that shows both the acceleration and the ECG signals while the acquisition is in progress[1]. The data were analyzed by using software developed in Matlab (The MathWorks, Inc). A peak detection algorithm, synchronized with the ECG, scans the first 150 ms following the R wave to locate the first heart sound vibration. Subsequently, the interval between the first heart sound and the following R wave is analyzed to record the amplitude (nadir to peak) of second heart sound vibration for each cardiac beat [3]. The accelerometer simply records naturally generated heart vibrations, which audible components give rise to the second heart sound 'See additional file 1: Appendix'.

**Figure 2:**
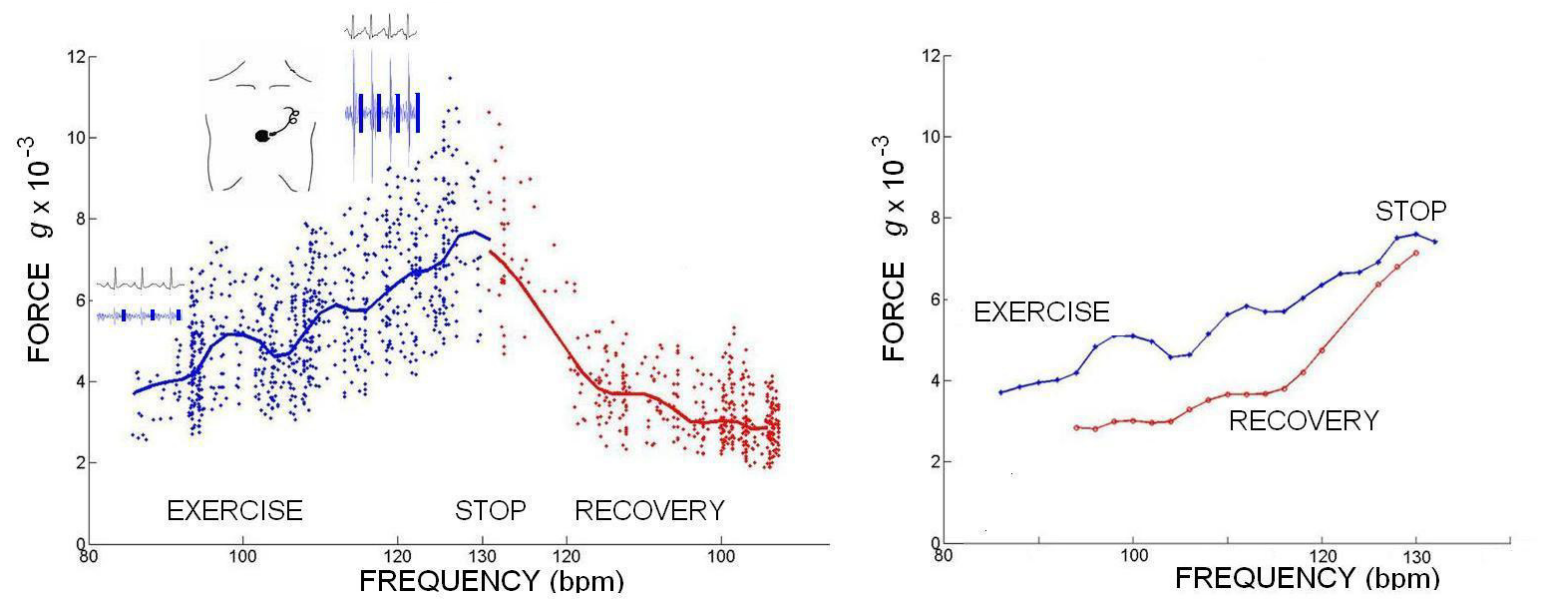
**Computing the second heart sound amplitude variation as a function of heart rate**. All the parameters are acquired as instantaneous values at baseline and during stress; mobile mean is utilized to assess baseline value (1 minute recording), at each incremental stress test, at peak test, and during recovery. Left panel: instantaneous S2 amplitude scattering (blue points exercise, red points recovery) depends on the respiratory cycle and thorax expansion; blue and red curves = S2 amplitude mobile mean. Right panel: blue curve = exercise in progress; red curve = recovery.

The curve of S2 peak amplitude variation as a function of heart rate was finally computed as the increment with respect to the resting amplitude value [13]. All the parameters were acquired as instantaneous values at baseline and during stress; mobile mean was utilized to assess baseline value (1 minute recording), at each incremental stress test, at peak test, and during recovery (Figure 2). Baseline, peak stress, peak-rest difference as absolute value, and delta % rest-peak stress values were computed.

Non myocardial noising vibrations (skeletal muscles, body movements, breathing) were eliminated by frequency filtering.

## Statistical analysis

SPSS 11 for Windows was utilized for statistical analysis. The statistical analyses included descriptive statistics (frequency and percentage of categorical variables and mean and standard deviation of continuous variables).

The one-way ANOVA was used to compare continuous variables between groups; when homogeneity of variance was not present, the Kruskal-Wallis test for nonparametric independent samples was used. Intergroup comparison was performed with Scheffe and Tamhane post hoc tests, respectively.

Relations between variables were assessed using linear regression analysis and Pearson's correlation coefficient. Cardiac or vascular properties significantly related to the S2 amplitude changes were graphically displayed with simple scatter plots. Crosstabs' statistics and measures of association for post exercise hypotension vs. post exercise S2 amplitude undershoot were performed in 75 selected patients.

## Results

### Resting and stress echocardiographic findings

Technically adequate images were obtained in all patients at baseline (by selection) and during stress.

#### At Peak Exercise

Heart rate was lower in the dipyridamole than in the exercise and pacing groups. The mean ejection fraction increased in the exercise and Dip groups, while decreased in the pacing group. Regional wall motion abnormalities occurred in 5 patients of the exercise, 1 patient of Dip and 2 patients of the pacing groups (Table 2).

**Table 2:** Rest and stress data

	EXERCISE		DIP		PACING
N of pts	99		41		6
Age (yrs)	56 ± 14	§	68 ± 11		68 ± 10
Gender (M/F)	68/31		27/14		4/2
BSA (m^2^)	1.88 ± .19		1.83 ± .16		1.87 ± .28
**Standard echo measurements**					
LVMI (g/m^2^)	104 ± 28		104 ± 20		138 ± 34
HR rest (bpm)	73 ± 16		66 ± 13		71 ± 10
HR peak (bpm)	131 ± 24	Δ	84 ± 13	*	132 ± 13
LV EF % rest	59 ± 11		58 ± 13		51 ± 11
LV EF % peak	67 ± 14	‡	62 ± 13	*	45 ± 16
WMSI rest	1.11 ± .29		1.17 ± .32		1.28 ± .46
WMSI peak	1.13 ± .31		1.19 ± .32		1.4 ± .46
Δ WMSI (rest-peak)	.02 ± .10		.01 ± .07		.15 ± .24
**Sensor built second heart sound (S2) amplitude changes**					
S2 rest (m*g*)	7.7 ± 4.9		7.1 ± 2.8		5.8 ± 1.4
S2 peak (m*g*)	15.9 ± 8.7	§	7.2 ± 3		7.7 ± 2.4
S2 Δ rest-peak (m*g*)	8.2 ± 6.1	§	.1 ± 1.5		1.8 ± 1.9
S2 Δ % (rest-peak)	133 ± 104	§	2 ± 22		31 ± 27
**Perpheral pressures, load and coupling**					
SBP rest (mmHg)	134 ± 21		137 ± 20		131 ± 25
SBP peak (mmHg)	189 ± 26	§	127 ± 26		137 ± 37
Δ SBP (rest-peak, mmHg)	55 ± 25	§	-8 ± 17		6 ± 17
DBP rest (mmHg)	74 ± 12		71 ± 12		74 ± 11
DBP peak (mmHg)	94 ± 13	§	67 ± 13		75 ± 15
Δ DBP (rest-peak, mmHg)	20 ± 13	§	-4 ± 10		1 ± 15
Mean pressure rest (mmHg)	94 ± 13		93 ± 12		93 ± 14
Mean pressure peak (mmHg)	126 ± 15	§	88 ± 17		96 ± 20
Δ mean pressure (rest-peak, mmHg)	32 ± 14	§	-5 ± 12		2 ± 15
SVR rest (dyne * sec * cm^-5^)	2134 ± 802		2118 ± 702		1652 ± 533
SVR peak (dyne * sec * cm^-5^)	1501 ± 547		1551 ± 747		1546 ± 620
Δ SVR (rest-peak, dyne * sec * cm^-5^)	-632 ± 669	‡	-567 ± 613		-106 ± 382
Arterial compliance rest (mL *m^-2^/mmHg)	0.49 ± 0.18		0.48 ± 0.2		0.7 ± 0.38
Arterial compliance peak (mL *m^-2^/mmHg)	0.33 ± 0.11	Δ	0.55 ± 0.22		0.4 ± 0.2
Δ Arterial compliance (rest-peak, mL *m^-2^/mmHg)	-0.17 ± 0.17	Δ	0.07 ± 0.15	*	-0.3 ± 0.24
Arterial elastance index rest (mmHg/mL/m^2^)	4.7 ± 1.5		4.5 ± 1.5		3.6 ± 1.1
Arterial elastance index peak (mmHg/mL/m^2^)	6.2 ± 1.8	Δ	4.1 ± 1.1	*	6.3 ± 2.5
Δ Arterial elastance index (rest-peak, mmHg/mL/m^2^)	1.5 ± 1.6	Δ	-.4 ± 1.4	*	2.7 ± 1.7
Ventricular/arterial coupling rest (SP/ESV/EaI ratio)	1.8 ± .9		1.8 ± .9		1.3 ± .7
Ventricular/arterial coupling peak (SP/ESV/EaI ratio)	2.9 ± 1.9	§	2.1 ± 1.1		1.1 ± .8
Δ Ventricular/arterial coupling (rest-peak)	1.1 ± 1.6	§	.4 ± .6		-0.2 ± .4
Cardiac index rest (L/min/m^2^)	2 ± 0.7		1.9 ± 0.5		2.5 ± 0.7
Cardiac index peak (L/min/m^2^)	3.9 ± 1.3	§	2.7 ± 0.9		2.7 ± 0.8
Δ Cardiac index (rest-peak, L/min/m^2^)	1.9 ± 1.2	§	0.7 ± 0.6		0.3 ± 0.5

§= significant differences between exercise and both dipyridamole and pacing stress pts; ‡ = significant differences between exercise and pacing stress pts; * = significant differences between dipyridamole and pacing stress pts; Δ = significant differences between exercise and dipyridamole stress pts

#### Peripheral pressures, load and coupling

Arterial elastance increased in the exercise and the Pacing groups, while decreased in the dipyridamole, mainly due to a greater dipyridamole induced arterial compliance (Table 2).

Despite similar baseline values, diastolic blood pressure increased in the exercise, decreased in the dipyridamole, while unchanged in the pacing group, although the response was heterogeneous at the individual level (Table 2).

### Sensor built second heart sound amplitude variations

**Figure 3:**
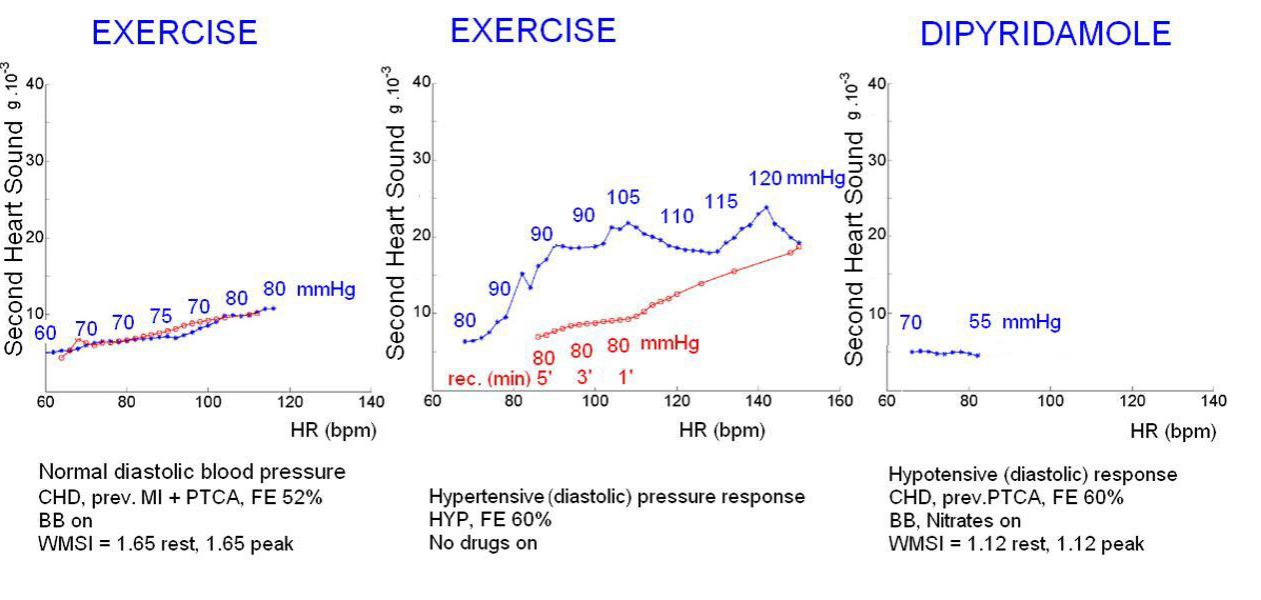
**Second heart sound (S2) amplitude recording simultaneously with diastolic blood pressure during stress**. Left panel: similar S2-frequency trend during stress (blue symbols) and recovery (red symbols) in a patient with normal exercise pressure changes and without post exercise hypotension. Middle panel: S2-frequency trend during stress (blue symbols) and recovery (red symbols) in a patient with exercise induced diastolic hypertension and post exercise hypotension. Right panel: flat-negative S2-frequency trend during dipyridamole stress induced hypotension.

A consistent second heart sound signal was obtained in all patients at rest and during stress (Figure 2). In the patients as a whole, baseline S2 was 7.2 ± 3.3 m*g*, increasing to 12.7 ± 7.7 m*g *at peak stress. S2 trends during exercise or dipyridamole are shown in Figure 3.

Mean S2 percentage increase was + 133 ± 104% in the 99 exercise patients, + 2 ± 22% in the 41 dipyridamole patients and + 31 ± 27% in the 6 pacing patients (p < 0.05 between groups) (Table 2).

In the exercise group the S2 amplitude percentage increase was similar in the 13 control and in the 86 patients (+ 140 ± 123% vs. 132 ± 102%, p = ns)

**Figure 4:**
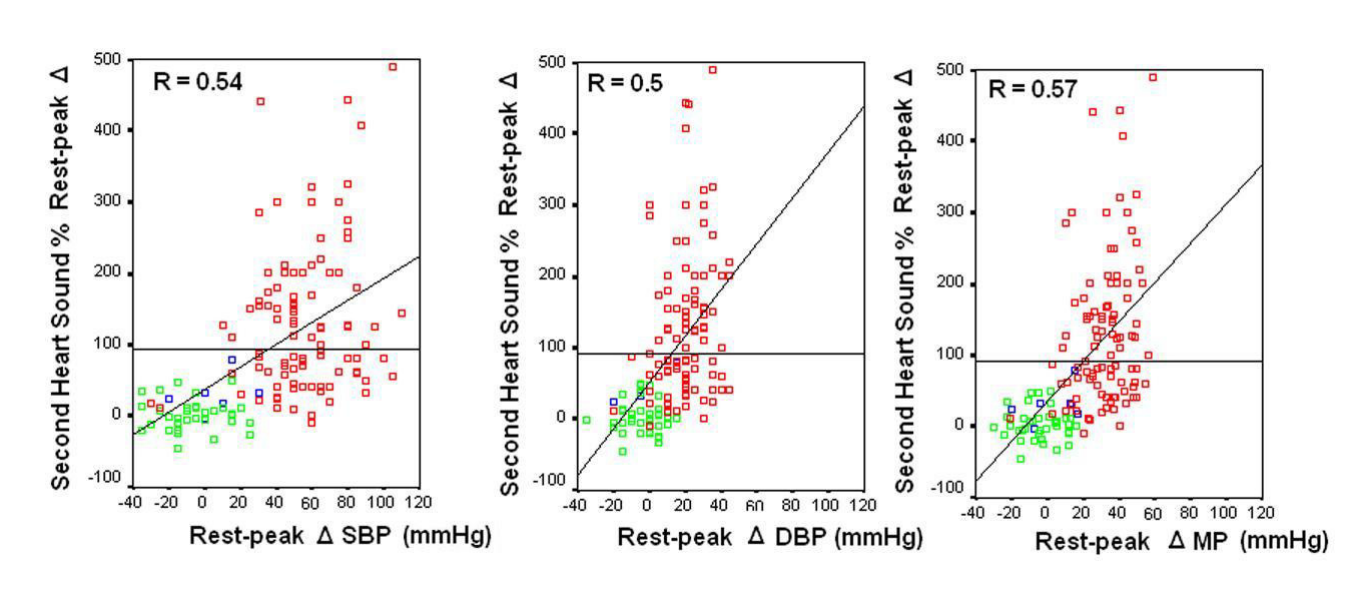
**Second Heart sound recording quantitatively documents systemic pressure changes**. Scatter plots demonstrating relationship between sensor Second Heart Sound amplitude % changes (y axis) and systemic pressure rest-peak changes values (x axis) in the whole group of 146 patients. Red symbols: exercise stress; green symbols: dipyridamole stress; blue symbols: pacing stress. Left panel: systolic pressure (SBP) changes. Middle panel: diastolic pressure (DBP) changes. Right panel: mean pressure (MP) changes.

At linear regression analysis significant positive determinants of the S2 amplitude changes during stress were the systemic blood pressure, the heart rate, and cardiac index rest-peak changes (Table 3). Scatter plots demonstrating correlations between S2 changes and arterial pressure rest-peak changes are displayed in Figure 4.

**Table 3:** Significant determinants of the sensor second heart sound (S2) amplitude values

	Rest S2	Peak S2	S2 Δ % rest-peak
Age (yrs)	-.359 (<.01)	-.476 (<.01)	-.153 (<.05)
BSA (m2)			
LVMI (g/m2)	-.194 (<.05)		
LV EF %		.215 (<.01)	
WMSI			
HR (bpm)	.206 (<.01)	.516 (<.01)	.453 (<.01)
Diastolic Blood Pressure (mmHg)	.183 (<.05)	.319 (<.01)	.502 (<.01)
Systolic Blood Pressure (mmHg)		.338 (<.01)	.544 (<.01)
Mean Blood Pressure (mmHg)		.345 (<.01)	.567 (<.01)
Ventricular elastance (mmHg/mL/m2)		.144 (<.05)	.218 (<.01)
Arterial elastance		.307 (<.01)	.281 (<.01)
SVR (dyne * sec * cm^-5^)			
Arterial compliance (mL *m^-2^/mmHg)		-.340 (<.01)	-.300 (<.01)
Ventricular/arterial coupling			
Cardiac index	.153 (<.05)	.432 (<.01)	.388 (<.01)

Linear regression analysis to identify significant relationship between predictor variables (first column) and the sensor second heart sound (S2) amplitude was performed for baseline (second column) peak stress (third column) and rest-peak delta values (fourth column).

Pearson's correlation coefficients (and significance value within brackets) are reported in cells with significant (p < 0.05) relationships.

### Second heart sound undershoot and the post exercise hypotension

A significant correlation was found between post exercise hypotension and recovery S2 undershoot: 44 (80%) of the 55 patients with post-exercise hypotension had S2 undershoot in the recovery, while 19 (96%) of the 20 patients without post-exercise hypotension had stable rate-S2 curve at recovery (Table 4) (Figure 3).

**Table 4:** Crosstabs' statistics and measures of association for post exercise hypotension vs. post exercise second heart sound amplitude undershoot in 75 selected patients

	Exercise recovery hypotension	Exercise recovery isopressure	Total
SHS Recovery Under shot	**44**	**1**	45
SHS Recovery same shot	**11**	**19**	30
Total	55	20	75

Kendall's tau-c = 0.591 P < 0.001

## Discussion

A stable, reproducible, and consistent S2 force signal was recorded in all patients at rest and during stress. Baseline force value had an ample range (from 2 to 23 g * 10^-3^). The most widely accepted theory for the genesis of the second heart sound is the "cardiohemic model," which states that the sounds are produced by the vibration of the entire heart and its contents [14]. This vibration is triggered by valve closure (the aortic and pulmonary valves for the second heart sound). The amplitude of these sounds depends on the force with which the valves close, which, in turn, depends on the pressure gradient across the valve at the time of closure. We previously demonstrated that in adult patients undergoing stress testing, the first heart sound amplitude was directly related to myocardial contractility [1]. In this investigation, blood pressure (systolic, diastolic and mean) correlated closely with S2 amplitude. This may be explained by the fact that amplitude is primarily determined by one factor, the force of valve closure [15].

### Biophysics of the second heart sound

Early studies of the hemodynamic determinants of the amplitude of the S2 have related the aortic component amplitude of the S2 vibration to the aortic pressure, in agreement with clinical findings that hypertensive patients frequently have loud second heart sounds [4,16,17]. In their proposed mechanism for the origin of the second heart sound, Sabbah and Stein [6] showed a relation between the amplitude of S2 and the driving pressure. Driving pressure, in the heart, refers to the instantaneous difference between arterial and ventricular pressure shortly after semilunar closure. Kusukawa and associates [5] previously found a good correlation of the amplitude of the second heart sound with the difference of pressure between the aorta and the left ventricle coincident with the dicrotic notch. But patients suffering from myocardial infarction and/or heart failure, often exhibit reduced S2 amplitude, even when the aortic pressure is normal [18]. They showed that the amplitude of S2 was linearly related to the rate of change of the pressure gradient that develops across the aortic valve during diastole (r = .82). The latter is also correlated with negative dP/dt (r = .62).

In normotensive patients with poor ventricular performance, the rate of isovolumic relaxation may be compromised and this would cause a reduction in negative dP/dt which in turn causes a reduction of the rate of change of the pressure gradient that develops across the valve during diastole. A diminished S2, therefore, would result due to the more slowly developing driving pressure, which directly affects the characteristics of valvular vibration. Tanigawa et al [4] demonstrated in instrumented dogs, that when the time constant of left ventricular pressure fall "T" was normal, the aortic systolic pressure and diastolic pressure were good predictors of S2 intensity. When LV relaxation was impaired, increasing T greater than 135% of control, the S2 intensity for any given aortic pressure was reduced. When relaxation was hyperactive, decreasing T less than 65% of control, S2 intensity was increased. Aortic pressure/T which assessed both aortic pressure and relaxation ability, is a better determinant of A2 intensity than aortic systolic pressure or aortic diastolic pressure alone.

### Second heart sound frequencies or amplitude to get clinical information?

Previous studies have shown that it is possible to estimate systemic blood pressure using the spectral information of the second heart sound. A mathematical model for the vibration of the closed aortic valve was proposed by Zhang et al [19], showing that the increasing aortic pressure results in an increase both in frequency and amplitude of produced sound. The results of this study also suggest that it is the increasing resonant frequency and amplitude of the blood column induced by elevated distending pressure that plays significant role in the process.

Various mathematical methods have been used to describe heart sounds, including the frequency domain (FFT) and the time domain (RMS) amplitude.

### The frequency domain (FFT)

The frequencies present in heart sounds are determined by the volume of the vibrating mass (smaller volume has a higher resonance frequency) and the tension generated in the walls of the heart and great vessels. This explains the fact that S2 is normally of higher frequency than S1 (the aorta is of lower volume than the heart) and that younger children exhibited higher heart sound frequencies than older children [20]. Other Authors [21] stated that the major concentration of energy, for both first heart sound (M1) and second heart sound (S2), is below 150 Hertz (Hz) which may indicate that both sounds are caused by vibrations within the same structure, possibly the entire heart. However S2 spectra have greater amplitude than S1 spectra above 150 Hz, which may be due to vibrations within the aorta and pulmonary artery. Because peak frequency is a descriptor of only a single point, it is therefore not a useful factor in describing heart sound changes resulting from variations in myocardial contractility or systemic pressure changes 'See additional file 1: Appendix'.

### The amplitude domain (RMS)

In previous investigation [20], hemodynamic variables (heart rate and blood pressure) correlated more closely with amplitude than with frequency. This may be explained by the fact that amplitude is primarily determined by one factor – force of valve closure – whereas frequency depends on the force of closure, heart volume, and the resonance frequencies of the heart and great vessels. Thus, differences in heart size and intravascular volume status could explain the greater variability (and, thus, weaker statistical correlation) in frequency than amplitude characteristics.

This is the motive for we used a peak amplitude (nadir to peak) signal analysis system for both the first and the second heart sounds vibrations [1,3].

### The properties of the chest wall in the transmission of sound from inside the thorax to the surface of the chest

The chest wall is a low-pass filter. Cardiac vibrations propagate as mechanical shear waves, and the intervening viscoelastic thoracic tissue attenuates the higher frequencies and introduces a variable propagation delay [22,23].

In contrast to the dynamics observed epicardially, Wood [24] demonstrated that heart sound frequency law was dominated by quasi-stationary and impulse-like components implying that the instantaneous power and the power spectrum contain most of the diagnostic information in heart sound.

Modelling the heart/thorax acoustic system in dogs, based on the simultaneous recording of the intracardiac and thoracic phonocardiograms, Durand and co-workers [25] showed that the heart/thorax acoustic system acts like a band pass filter having a higher attenuation for A2 than for M1. Between 20 and 100 Hz, the mean attenuation of M1 is 30 dB while that of A2 is 46 dB. Above 100 Hz, the attenuation slope is -12 dB per octave for M1 and -6 dB per octave for A2. Again, the frequency domain is influenced by the heart/thorax acoustic system, and the frequency based heart sound information is jeopardized by a further variable. Using heart sound amplitude to get clinical information, the absolute force value in the single patient is certainly related to the transthoracic propagation of cardiac vibrations. In fact, when measured epicardially or on the aortic root, S2 vibrations are up to 10 times more powerful than when measured on the chest, and cannot be used as absolute value for interpatient comparison. However the amplitude (force) % changes (i.e. contractility for M1 and systemic pressure for S2) are not influenced by the heart/thorax acoustic system and the data can be used for intrapatient changes as for contractility or systemic pressure changes.

### Second heart sound and stress changes

Previous phonocardiography research has been focused on the determination of heart sound production at rest, but relatively little work has been done to investigate heart sounds under stress testing. Luisada et al. [26] stated that heart sound changes during stress may be more rapid and sensitive than changes in heart rate and blood pressure. Of the 146 study patients, 39 unchanged or decreased diastolic blood pressure at peak stress vs. rest (mainly dipyridamole group, 28 out of 41 pts) while 107 increased diastolic blood pressure (mainly exercise group, 91 out of 99 pts). Patients with increased pressure had + 116 ± 106% second heart sound amplitude increase vs. + 26 ± 67% in patients with unchanged or decreased diastolic blood pressure at peak stress. In our study, a mismatch between increased diastolic pressure, but blunted S2 amplitude, occurred in 7 patients out of the 107 with stress increased diastolic pressure. According to the physiological basis, in these case the blunted S2 increase should be related to a diminished driving pressure between the aorta and the left ventricle, with delayed or altered active LV relaxation. These 7 patients had coronary artery disease. Obviously, sensor measured S2 amplitude, without ventricular relaxation data, blind us to the quantification of the time constant of left ventricular pressure fall, and/or to negative LV dP/dt. However, this totally noninvasive sensor demonstrated capability to monitor beat to beat systemic pressure changes, at rest and during exercise. Further studies with simultaneous hemodynamic in humans should be done to address this issue.

### Second heart sound and post exercise hypotension

Post exercise hypotension has been demonstrated both in hypertensive and healthy subjects [27] . In normotensive subjects, it has been attributed to a decrease in cardiac output and/or systemic vascular resistance [28,29]. Moreover, it has been accompanied by a decrease in peripheral sympathetic activity [29,30] and an increase in cardiac sympathetic activity [31]. Other studies demonstrated that the acute post-exercise reduction in blood pressure was clinically similar following high intensity short duration exercise and moderate intensity longer duration exercise [32]. Acute exercise may serve as a non-pharmacological aid in the treatment of hypertension. S2 amplitude monitoring could be a method to assess efficacy of the acute post-exercise blood pressure reduction. In the selected patients of our study, a significant correlation was found between post exercise hypotension and recovery second heart sound lower amplitude, to confirm the capability of the sensor to mirror diastolic pressure trend.

## Limitations of the study

We used intermittent auscultatory methods to determine exercise and post-exercise blood pressure. These auscultatory methods are prone to sampling error and may provide inaccurate results. Since diastolic isovolumic relaxation occurs simultaneously with the physical phenomenon (the abrupt deceleration of the moving aortic blood mass), that gives rise to the S2 amplitude, the S2 amplitude is an algebraic sum of the myocardial and of the aortic blood mass effects. Several scenarios can occur for S2 amplitude. 1 – With constant ventricular relaxation rate, S2 amplitude is directly related to the diastolic aortic pressure: 2 – With constant aortic diastolic pressure, S2 amplitude is directly related to the ventricular relaxation rate. Obviously, sensor measured S2 amplitude, without ventricular relaxation hemodynamics, cannot sense the ventricular component of the S2. Further studies in humans with simultaneous hemodynamic assessment should be done to address this issue. Another limitation of the study could arise from the fact that we didn't measure the split in the second cardiac sound [33,34]. The continuous wavelet transforms (CWTs) method is capable of detecting its two components, A2 and P2, allowing therefore the measurement of the delay between them. This delay, called the split, is very important in the diagnosis of many pathological cases, but it was not the aim of this study

### Characteristics of the population and inducible ischemia

Eight (5%) of the 146 patients had stress induced ischemia. The low rate of test positivity depends on many factors. The test indication class was not always I or IIa: low appropriateness in a high volume laboratory setting mainly depends on too often repeated tests in the absence clinical changes [35]. Second, stress test was often performed in young patients with low pre-test probability of CAD (13 controls and 39 patients with atypical chest pain and/or systemic hypertension). Third, valvular heart disease patients (moderate aortic stenosis in 9, moderate mitral regurgitation in 10) were referred for Doppler stress echo. Fourth, 17 CAD patients underwent dipyridamole stress for coronary flow reserve evaluation of left anterior descending coronary artery [8].

## Conclusion

Continuous and non-invasive monitoring of blood pressure (BP) is important to prevent hypertensive patients from stroke and heart attack. However, most of the prevalent BP devices can provide solely intermittent measurements. S2 recording quantitatively documents systemic pressure changes: S2 amplitude trend is up-sloping when pressure increases as may occur during physical exercise or is flat for a flat pressure trend as may occur during dipyridamole induced vasodilatation. A new concept of non-invasive blood pressure measurement by heart sound pattern analysis is described. The known diagnostic criterion of the 'accentuated' second heart sound of a hypertensive patient is here converted into a computer-aided pattern-recognition process for the second heart sound, applicable over the entire range of blood pressure. The method is in principle suited for automatically repeated blood pressure measurements, but further development is still needed for conversion into a widely practicable procedure. Integrating first heart sound [1], second heart sound amplitude and first-second heart sound time delay [3], a cutaneous operator-independent force sensor describes in real time systolic elastance, diastolic time, and systemic pressure trend, offering a new chance to monitor failing hearts.

## Abbreviations

A2: aortic component of the second heart sound; BSA: body surface area; C: systemic arterial compliance; DBP: diastolic blood pressure; CAD: coronary artery disease; CO: cardiac output; DCM: idiopathic dilated cardiomyopathy; EaI: effective arterial elastance index; EDV: end-diastolic volume; EF: ejection fraction; ESV: end-systolic volume; FFR: force-frequency relation; *g*: acceleration unit (9.8 m/sec^2^); HR: heart rate; LV: left ventricle/ventricular; LVMI: left ventricular mass index; M1: mitral component of the first heart sound; P2: pulmonary component of the second heart sound; S1: first heart sound; S2: second heart sound; SBP: systolic blood pressure; SVR: systemic vascular resistance; WMSI: wall motion score index.

## Competing interests

The authors declare that they have no competing interests.

## Authors' contributions

TB conceived this study, performed the data analysis, and drafted the manuscript; LV, CP, EPa, LP and MP were responsible for data collection and revised the manuscript; VG, EB, FF and MG were responsible for technology development and digital signal processing; GA gave a contribution to data discussion; EPi gave a contribution to preparation of study design, data discussion, and critical revision of the manuscript.

## Supplementary Material

Additional file 1 Appendix. Sound – Heart sounds – Accelerometer to measure peak heart sounds vibration amplitude – Wireless – Wireless telemedicine – Telemedicine is healthcare's new frontier. Click here for file
