# Supplementary material for: Arterial pressure changes monitoring with a new precordial noninvasive sensor
Source: Cardiovasc Ultrasound. 2008 Aug 21;6:41. doi: 10.1186/1476-7120-6-41 (PMC2531180; doi:10.1186/1476-7120-6-41)
Supplement: Additional file 1 — Appendix. Sound – Heart sounds – Accelerometer to measure peak heart sounds vibration amplitude – Wireless – Wireless telemedicine – Telemedicine is healthcare's new frontier. [file 1476-7120-6-41-S1.doc]

**Appendix**

**Sound**

Sound is [vibration](http://en.wikipedia.org/wiki/Vibration) transmitted through a [solid](http://en.wikipedia.org/wiki/Solid), [liquid](http://en.wikipedia.org/wiki/Liquid), or [gas](http://en.wikipedia.org/wiki/Gas) as [longitudinal waves](http://en.wikipedia.org/wiki/Longitudinal_wave), also called [compression](http://en.wikipedia.org/wiki/Compression) waves. Longitudinal sound waves are waves of alternating [pressure](http://en.wikipedia.org/wiki/Pressure) deviations from the [equilibrium](http://en.wikipedia.org/wiki/Equilibrium) pressure, causing local regions of [compression](http://en.wikipedia.org/wiki/Physical_compression) and [rarefaction](http://en.wikipedia.org/wiki/Rarefaction). Matter in the medium is periodically displaced by a sound wave, and thus oscillates. Particularly, sound means those vibrations composed of [frequencies](http://en.wikipedia.org/wiki/Frequencies) [capable of being detected by ears](http://en.wikipedia.org/wiki/Threshold_of_hearing). For humans, hearing is limited to frequencies between about 20 [Hz](http://en.wikipedia.org/wiki/Hertz) and 20,000 Hz (20 [kHz](http://en.wikipedia.org/wiki/KHz)), with the upper limit generally decreasing with age.

***Sound properties*** Sound waves are characterized by the generic [properties of waves](http://en.wikipedia.org/wiki/Wave#Physical_description_of_a_wave), which are [frequency](http://en.wikipedia.org/wiki/Frequency), [wavelength](http://en.wikipedia.org/wiki/Wavelength), [period](http://en.wikipedia.org/wiki/Periodicity), [amplitude](http://en.wikipedia.org/wiki/Amplitude), [intensity](http://en.wikipedia.org/wiki/Intensity_(physics)), [speed](http://en.wikipedia.org/wiki/Speed_of_sound), and [direction](http://en.wikipedia.org/wiki/Direction_(geometry%2C_geography)) (sometimes speed and direction are combined as a [velocity](http://en.wikipedia.org/wiki/Velocity) [vector](http://en.wikipedia.org/wiki/Vector_(spatial)), or wavelength and direction are combined as a [wave vector](http://en.wikipedia.org/wiki/Wave_vector)).

***Frequency*** Frequency is a [measure](http://en.wikipedia.org/wiki/Measurement) of the number of occurrences of a repeating event per unit [time](http://en.wikipedia.org/wiki/Time).The hertz (symbol: Hz) is a measure of [frequency](http://en.wikipedia.org/wiki/Frequency), informally defined as the number of events occurring per [second](http://en.wikipedia.org/wiki/Second). It is the [basic unit](http://en.wikipedia.org/wiki/SI_base_unit) of [frequency](http://en.wikipedia.org/wiki/Frequency) in the [International System of Units](http://en.wikipedia.org/wiki/International_System_of_Units) (SI), and is used worldwide in both general-purpose and scientific contexts. *Hertz* can be used to measure any periodic event. When the loudness of a sound wave changes, so does the amount of compression in airwave that is traveling through it, which in turn can be defined as [amplitude](http://en.wikipedia.org/wiki/Amplitude).

***Amplitude*** Amplitude is the [magnitude](http://en.wikipedia.org/wiki/Magnitude_(mathematics)) of change in the oscillating variable, with each [oscillation](http://en.wikipedia.org/wiki/Oscillation), within an oscillating system. For instance, [sound waves](http://en.wikipedia.org/wiki/Sound_waves) are oscillations in [atmospheric pressure](http://en.wikipedia.org/wiki/Atmospheric_pressure) and their amplitudes are proportional to the change in [pressure](http://en.wikipedia.org/wiki/Pressure) during one oscillation. If a graph of the system is drawn with the oscillating variable as the vertical axis and time as the horizontal axis then the amplitude may be measured as the vertical distance between points on the curve. Peak-to-peak amplitude is to measure it between peak and trough. Peak-to-peak amplitudes can be measured by [meters](http://en.wikipedia.org/wiki/Measuring_instrument) with appropriate circuitry, or by viewing the waveform on an [oscilloscope](http://en.wikipedia.org/wiki/Oscilloscope), or by an accelerometer

***Speed*** The speed of sound depends on the medium through which the waves are passing, and is often quoted as a fundamental property of the material. In general, the speed of sound is proportional to the square root of the ratio of the [elastic modulus](http://en.wikipedia.org/wiki/Elastic_modulus) (stiffness) of the medium to its [density](http://en.wikipedia.org/wiki/Density).

The study of sound and vibration are closely related. Sound, or "pressure waves", are generated by vibrating structures; these pressure waves can also induce the vibration of structures.

**Heart sounds**

The heart sounds are the noises ([sound](http://en.wikipedia.org/wiki/Sound)) generated by the beating [heart](http://en.wikipedia.org/wiki/Heart) and the resultant flow of blood through it. The frequencies present in heart sounds are determined by the volume of the vibrating mass (smaller volume has a higher resonance frequency) and the tension generated in the walls of the heart and great vessels. This explains the fact that S2 is normally of higher frequency than S1 (the aorta is of lower volume than the heart) and that younger children exhibited higher heart sound frequencies than older children.

Various mathematical methods have been used to describe heart sounds. The time domain (RMS amplitude) and the frequency domain (FFT) methods have both proven useful, as a possible indicator of several heart and valve diseases.

Amplitude is primarily determined by one factor—force of valve closure—whereas frequency depends on the force of closure, heart volume, and the resonance frequencies of the heart and great vessels. Thus, differences in heart size and intravascular volume status could explain the greater variability (and, thus, weaker statistical correlation) in frequency characteristics than amplitude.

**Accelerometer to measure peak heart sounds vibration amplitude**

An accelerometer is a device for measuring [acceleration](http://en.wikipedia.org/wiki/Acceleration) and gravity induced reaction forces. Single- and multi-axis models are available to detect magnitude and direction of the acceleration as a vector quantity. Accelerometers can be used to sense inclination, vibration, and shock. They are increasingly present in portable electronic devices. An accelerometer measures the [acceleration](http://en.wikipedia.org/wiki/Acceleration) and gravity it experiences. Both are typically expressed in SI units [meters/second^2^](http://en.wikipedia.org/wiki/Metre_per_second_squared) (m·s^2^) or popularly in terms of [g-force](http://en.wikipedia.org/wiki/G-force). The effects of gravity and acceleration are indistinguishable, following [Einstein's equivalence principle](http://en.wikipedia.org/wiki/Equivalence_principle). As a consequence, the output of an accelerometer has an offset due to local gravity. This means that, perhaps counter-intuitively, an accelerometer *at rest* on the earth's surface will actually indicate 1 g along the vertical axis. To obtain the acceleration due to motion alone, this offset must be subtracted. Along all horizontal directions, the device yields acceleration directly. For the practical purpose of finding the acceleration of objects with respect to the earth, such as for use in an inertial navigation system, the correction due to gravity along the vertical axis is usually made automatically, e.g. by calibrating the device at rest. Modern accelerometers are often small *micro electro-mechanical systems* ([MEMS](http://en.wikipedia.org/wiki/Microelectromechanical_systems)), and are indeed the simplest MEMS devices possible, consisting of little more than a [cantilever beam](http://en.wikipedia.org/wiki/Cantilever) with a proof mass (also known as seismic mass) and some type of deflection sensing circuitry. Under the influence of gravity or acceleration the proof mass deflects from its neutral position. The deflection is measured in an analog or digital manner. Single-axis, dual-axis, and triple-axis models exist to measure acceleration as a vector quantity or just one or more of its components. MEMS accelerometers are available in a wide variety of measuring ranges, reaching up to thousands of [*g*](http://en.wikipedia.org/wiki/G-force)*'*s. Accelerometers are increasingly being incorporated into personal electronic devices such as media players, gaming devices, or step counters. One of the most common uses for [MEMS](http://en.wikipedia.org/wiki/Microelectromechanical_systems) accelerometers is in [airbag](http://en.wikipedia.org/wiki/Airbag) deployment systems for modern automobiles. Another common automotive use is in [electronic stability control](http://en.wikipedia.org/wiki/Electronic_stability_control) systems, which use a lateral accelerometer to measure cornering forces.

***g-force*** *g*-force (also G-force, g-load) is a measurement of an object's [acceleration](http://en.wikipedia.org/wiki/Acceleration) expressed in *g*s. It is an indication of the reaction force resulting from this acceleration or, more correctly, the net effect of that acceleration and the acceleration imparted by natural [gravity](http://en.wikipedia.org/wiki/Gravitation) as subjectively experienced by an object. Note that *g*-force is a misnomer, because it is about acceleration (m/s^2^). [Force](http://en.wikipedia.org/wiki/Force) is the combined effect of [acceleration](http://en.wikipedia.org/wiki/Acceleration) and [mass](http://en.wikipedia.org/wiki/Mass) (kg*m/s^2^). G-force is, however, often used for the acceleration of one specific body of mass (such as a human body), so in that sense one could say it *does* quantify force, even though the unit used is that of acceleration (for a given mass).

The *g* is a non-[SI](http://en.wikipedia.org/wiki/International_System_of_Units) unit equal to the nominal acceleration of gravity on Earth at sea level ([standard gravity](http://en.wikipedia.org/wiki/Standard_gravity)), which is defined as 9.80665 m/s^2^ (32.174 ft/s^2^). The symbol *g* is properly written both lowercase and italic to distinguish it from the symbol *G*, the [gravitational constant](http://en.wikipedia.org/wiki/Gravitational_constant) and g, the symbol for [gram](http://en.wikipedia.org/wiki/Gram), a unit of mass, which is not italicized. Because of the potential for confusion about whether g-force measures acceleration or force, the term is considered by some to be a misnomer. Scientific usage prefers explicit reference to either acceleration or force, and use of the appropriate units (in the [SI](http://en.wikipedia.org/wiki/SI) system, [meters per second squared](http://en.wikipedia.org/wiki/Metre_per_second_squared) for acceleration, and [newtons](http://en.wikipedia.org/wiki/Newton) for force).

**Wireless**

Wireless communication is the transfer of *information* over a distance without the use of electrical conductors or "[wires](http://en.wikipedia.org/wiki/Wire)".The distances involved may be short (a few meters as in television remote control) or very long (thousands or even millions of kilometers for radio communications). When the context is clear the term is often simply shortened to "wireless". Wireless communications is generally considered to be a branch of [telecommunications](http://en.wikipedia.org/wiki/Telecommunications). Wireless operations permits services, such as long range communications, that are impossible or impractical to implement with the use of wires. The term is commonly used in the telecommunications industry to refer to telecommunications systems (e.g., radio transmitters and receivers, remote controls, computer networks, network terminals, etc.) which use some form of energy (e.g. [radio frequency](http://en.wikipedia.org/wiki/Radio_frequency) (RF), [infrared](http://en.wikipedia.org/wiki/Infrared) light, [laser](http://en.wikipedia.org/wiki/Laser) light, visible light, acoustic energy, etc.) to transfer information without the use of wires. Information is transferred in this manner over both short and long distances.

*Wireless communication* may be via:

- [radio](http://en.wikipedia.org/wiki/Radio) frequency communication,
- [microwave](http://en.wikipedia.org/wiki/Microwave) communication, for example long-range line-of-sight via highly directional antennas, or short-range communication, or

[infrared](http://en.wikipedia.org/wiki/Infrared) (IR) short-range communication, for example from [remote controls](http://en.wikipedia.org/wiki/Remote_control) or via [IRDA](http://en.wikipedia.org/wiki/IRDA),

**Wireless telemedicine**

Wireless telemedicine is a new and evolving area in telemedical and telecare systems. Healthcare personnel require real-time access to accurate patient data, including clinical histories, treatments, medication, tests, laboratory results and insurance information. With large-scale wireless networks and mobile computing solutions, such as cellular 3G, Wi-Fi mesh and WiMAX, healthcare personnel can tap into vital information anywhere and at any time within the healthcare networks. The recent introduction of pervasive computing, consisting of radio frequency identification (RFID), Bluetooth, ZigBee, and wireless sensor networks, further extends the potential for exploitation of wireless telecommunications and its integration into new mobile healthcare delivery systems.

Rapid advances in information technology and telecommunications, and more specifically wireless and mobile communications, and their convergence (telematics) are leading to the emergence of a new type of information infrastructure that has the potential of supporting an array of advanced services for healthcare.

# Telemedicine is healthcare’s new frontier

Telemedicine is healthcare's new frontier, a means of facilitating the distribution of human resources and professional competences. It can speed up diagnosis and therapeutic care delivery and allow peripheral and primary healthcare providers to receive continuous assistance from specialised centres. This is in response to the need to further tailor support for the development of telemedicine via satellite to comply with the key priorities of the healthcare system.

Some severe diseases and disorders e.g. diabetes and heart failure need close and continual monitoring procedure after diagnosis, in order to prevent mortality or further damage as secondary to the mentioned diseases or disorders. Monitoring these types of patients, usually, occur at hospitals or healthcare centres. However, the patients are often too early released, owing to need of hospital bed for another patient on the waiting list, who needs to be hospitalised immediately.

Long waiting time for hospitalisation or ambulatory patient monitoring/treatment, are other well-known issues for both the healthcare institutions and the patients. One of the primary challenges faced by healthcare authorities is being able to maximise the quality and breadth of healthcare services while controlling costs. As the population ages and demand for services increases, the ability to maintain the quality and availability of care, while effectively managing financial and human resources, is of critical importance. Use of modern communication technology in this context, is the sole decisive factor that makes such telemedicine system successful. Therefore, there is a strong need for investigating the possibility of design and implementation of an interactive real-time wireless telemedicine system. Early results showed that the system is reliable, functions with a clinically acceptable performance, and transfers medical data with a reasonable quality, even though the system was tested under totally uncontrolled circumstances during the patients’ daily activities. Thus, the system is applicable, and might be generalised in clinical practice e.g. in cardiology. Systems for intelligent remote monitoring of hearts could be developed. It is important for patients with chronic heart failure to be discharged from the hospital after an appropriate stabilization period for better recovery and quality of life. Reliable continuous remote monitoring systems for these patients are gaining practical meaning.
